# Experimental Control of Turbulent Boundary Layers with In-plane Travelling Waves

**DOI:** 10.1007/s10494-018-9926-2

**Published:** 2018-05-14

**Authors:** James Bird, Matthew Santer, Jonathan F. Morrison

**Affiliations:** Department of Aeronautics, Imperial College London, London, SW7 2AZ UK

**Keywords:** Flow control, Drag reduction, Turbulence, Adaptive structures

## Abstract

The experimental control of turbulent boundary layers using streamwise travelling waves of spanwise wall velocity, produced using a novel active surface, is outlined in this paper. The innovative surface comprises a pneumatically actuated compliant structure based on the kagome lattice geometry, supporting a pre-tensioned membrane skin. Careful design of the structure enables waves of variable length and speed to be produced in the flat surface in a robust and repeatable way, at frequencies and amplitudes known to have a favourable influence on the boundary layer. Two surfaces were developed, a preliminary module extending 152 mm in the streamwise direction, and a longer one with a fetch of 2.9 m so that the boundary layer can adjust to the new surface condition imposed by the forcing. With a shorter, 1.5 m portion of the surface actuated, generating an upstream-travelling wave, a drag reduction of 21.5*%* was recorded in the boundary layer with *Re*_*τ*_ = 1125. At the same flow conditions, a downstream-travelling produced a much smaller drag reduction of 2.6*%*, agreeing with the observed trends in current simulations. The drag reduction was determined with constant temperature hot-wire measurements of the mean velocity gradient in the viscous sublayer, while simultaneous laser Doppler vibrometer measurements of the surface recorded the wall motion. Despite the mechanics of the dynamic surface resulting in some out-of-plane motion (which is small in comparison to the in-plane streamwise movement), the positive drag reduction results are encouraging for future investigations at higher Reynolds numbers.

## Introduction

Finding new ways to improve the fuel efficiency of aircraft is undoubtedly a worthwhile endeavour for financial, social and environmental reasons. To continue such improvements, new technologies, currently in their infancy, need to be explored now to bring about step-changes in the aerodynamic drag reduction. One such ‘active’ strategy (requiring energy input external to the flow) is considered experimentally in this paper: the open-loop control of turbulent boundary layers with spanwise wall oscillations.

At flight speeds, the boundary layer over almost all of a transonic airliner fuselage is unavoidably turbulent, generating 50*%* of the total drag experienced by the aircraft [1]. The ubiquity of turbulent boundary layers, and their marked detrimental effect on aerodynamic efficiency, make them a potentially fruitful target for flow-control strategies.

Passive techniques can be employed to reduce turbulent skin-friction drag, such as the application of riblets [2] and super-hydrophobic surfaces [3] or polymer addition in liquids [4]. However, these schemes often deliver small benefits, or are unsuited to air. Recently, Ghebali et al. [5] explored a novel passive technique using an inclined wavy surface to bring about a drag reduction of 0.5*%* in a direct numerical simulation (DNS) of channel flow. The wavy walls drive spanwise momentum to emulate a spatial Stokes’ layer, and favourably alter the flow. Although the low spanwise velocities generated by the geometry, as well as the increased pressure drag due to its out-of-plane nature, limit the effectiveness of this approach, the beneficial spanwise velocity can be generated in other ways.

The influence of the spanwise velocity was first recognised by Bradshaw and Pontikos [6] who noted that the cross flow on an ‘infinite’ swept wing, and the resulting three-dimensionality of the boundary layer, led to a reduction in Reynolds shear stress. Downstream from the initial perturbation the flow inevitably returned to its original two-dimensional state. However, with the application of periodic forcing, these benefits can be continued indefinitely. In this way, the boundary layer is continuously perturbed ensuring it remains three-dimensional and never reaches an equilibrium state, the turbulence structures being forced to change in the streamwise direction [7].

The forcing can be applied directly, with Lorentz body forcing [8] or indirectly through an oscillating wall. The spanwise wall motion generates a Stokes’ layer, which then interacts with the turbulent boundary layer near the wall. The influence of the forcing has been investigated extensively in numerical and laboratory experiments, the consensus view being that, with the correct forcing parameters, a large reduction in skin friction can be produced. This phenomenon was first noted by Jung et al. [9] who carried out a DNS of channel flow at $Re_{\tau } = \frac {u_{\tau } d}{\nu }= 200$ (where *d* is the half-channel height, *u*_*τ*_ is the wall friction velocity of the uncontrolled channel and *ν* is the kinematic viscosity) and imposed an oscillating cross flow and also a periodic wall motion. A 40*%* reduction in skin friction drag was recorded when the forcing had a nondimensional time period $T^{+}=\frac {Tu_{\tau }^{2}}{\nu }= 100$. Quadrio and Ricco also performed a large number of DNS of turbulent channel flow at *Re*_*τ*_ = 200 with an oscillating wall [10]. They varied the of non-dimensional wall forcing period *T*^+^ and wall velocity $W^{+}=\frac {W}{u_{\tau }}$ and recorded changes in the wall shear stress and found an optimal forcing period of *T*^+^ ≈ 100 − 125 for drag reduction, which increased monotonically with wall velocity with a peak measured drag reduction of 44.7*%*. They also found a net power saving of 7.3*%* was possible, for an ideal actuator working solely against the frictional resistance of the flow. These findings, and similar levels of of drag reduction, have also been observed in other simulations of channel flow [7, 11].

Numerous experiments have also been performed, corroborating these findings. Laadhari et al. generated sinusoidal wall motion using a crank-slider system with a fixed displacement from 2 to 10 Hz [12]. Hot-wire measurements were then made in the turbulent boundary layer and the oscillations were found to produce a reduction in turbulent fluctuations. Similar experiments were carried out by Choi [13] and Gouder et al. [14], who developed electromagnetic and electro-active polymer surfaces: both measured similar reductions in turbulent fluctuations as well as skin-friction drag reductions of 45*%* and 17*%* respectively. Gatti et al. also conducted experiments using electro-active polymer surfaces and measured a drag reduction, integrated over the whole surface, of 2.4*%*. The work discussed in this paper differs from that of Gatti et al. by having a long and continuous fetch of wall motion under a developing boundary layer, rather than in fully developed channel flow. Importantly, the actuation mechanism explored in this work is different, and allows for waves of variable streamwise length to be produced, rather than a wall oscillation. It is important to note that the actuation method of Gatti et al. [15] was chosen for its efficiency, achieving only a very small net-power loss, while the goal of the work presented here is to achieve a drag reduction using travelling waves, irrespective of the energy input.

**Fig. 1:**
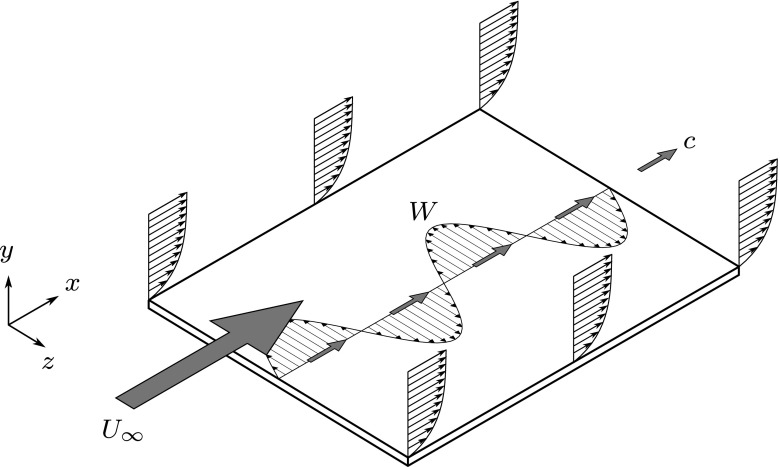
An illustration of the sinusoidal distribution of spanwise wall velocity *W*. The perturbation velocity is in the *z* direction, but its variation propagates in the streamwise direction, *x*, with velocity *c*

**Fig. 2:**
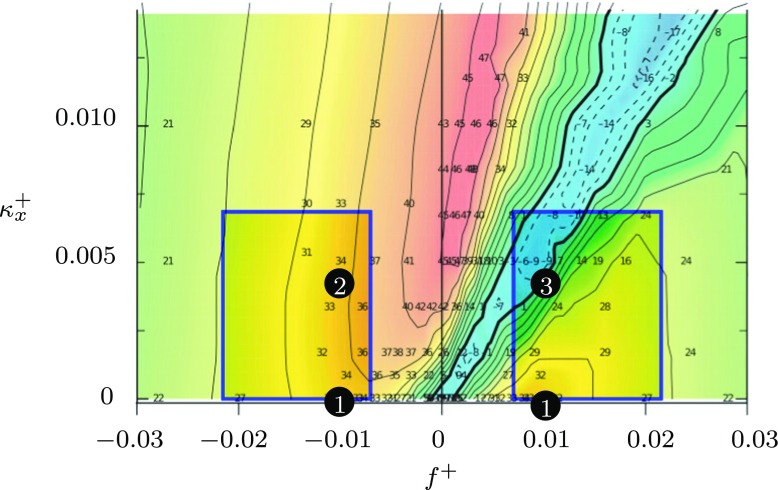
A map of drag reduction at *Re*_*τ*_ = 200 in a DNS carried out by Quadrio et al. [17] for a range of frequencies and wavenumbers. The blue boxes indicate the potential scope of the forcing in the present study, while the black dots indicate the measurement points

The wall motion discussed so far is a function of time and amplitude, but a spatially varying velocity distribution at the wall can yield additional benefits. Zhao et al. [16] have investigated how spanwise travelling waves of spanwise velocity can influence a turbulent boundary layer in DNS, achieving a peak drag reduction of about 30*%*. However, it is streamwise travelling waves of spanwise perturbation that offer the greatest potential, where the wall velocity *W* is distributed as

1 $$ W = A \sin\left( \kappa_{x}x - \omega t\right),  $$

and *A* is the forcing amplitude, *κ*_*x*_ is the streamwise wavenumber, *ω* is the frequency of oscillation, *x* is the streamwise distance and *t* is time. It is important to emphasise that the velocity of the motion (*W* ) is in the spanwise direction, while the velocity of the wave ($c=\frac {\omega }{\kappa _{x}}$) is in the streamwise direction. A wave of this nature under a boundary layer is illustrated in Fig. 1. Quadrio and Ricco [17] have performed numerous DNS of channel flow with *Re*_*τ*_ = 200, varying the parameters *κ*_*x*_ and *ω* in Eq. 1. For values of *κ*_*x*_ and *ω* which produced a wave moving slowly with the flow, they found a large drag reduction of 48*%* was achieved, compared to 34*%* for the wall oscillation case (*κ*_*x*_ = 0), for the same forcing amplitude *W*^+^. For upstream-travelling waves, they found that a drag reduction was always produced. Alternatively, if the wave speed (*c*) was similar to, and in the same direction as, the mean streamwise velocity of near wall (*y*^+^ < 10) structures, when *c* ≈ 10*u*_*τ*_, a large drag increase of up to 23*%* was generated. A map displaying some of these results for different forcing parameters is shown in Fig. 2. Quadrio and Ricco also found that as the maximum drag reduction occurs with parameter values which also require a minimum actuation effort, the streamwise travelling waves offer a unique potential of a large power saving [17]. For an ideal system, where the only losses are incurred from the spanwise work against the frictional resistance of the fluid, a power saving of 26*%* has been recorded compared to only 5*%* when the wall oscillation is not modulated spatially, for the same forcing amplitude.

Similar trends have been also been observed in boundary layer flows. Skote et al. performed DNS of temporally and spatially varying wall oscillation under a spatially developing boundary layer with $\displaystyle {Re_{\theta }=\frac {U_{\infty } \delta _{\theta }}{\nu }}$ ranging from 418 to 750, for the unforced condition, where *δ*_*𝜃*_ is the boundary layer momentum thickness, and *U*_*∞*_ is the freestream velocity [18, 19]. They also found a larger drag reduction of 41*%* for spatially modulated forcing compared to 29.4*%* for the wall oscillation case, with a wall velocity *W*^+^ = 12. At a lower actuation velocity of *W*^+^ = 6, a significant net power saving of 16.3*%* was measured for the spatial oscillation, whilst only a modest saving was recorded for the temporal oscillation of 2.85*%*. The values of drag reduction measured in these external flows are lower than that for channel flows, at similar Reynolds numbers and wall velocity. This is corroborated by Lardeau and Leschziner [20] who conducted simulations of a boundary layer with *Re*_*τ*_ = 520 just upstream of a wall oscillating with period *T*^+^ = 80 to 200. They found the maximum drag reduction occurred at a lower actuation period *T*^+^ < 80 than in channel flow (*T*^+^ = 100 - 120), and the extent of the reduction was 5-7*%* less.

To date, the majority of the experiments and simulations have been carried out at low Reynolds numbers, that is below *Re*_*τ*_ = 1000 and typically at *Re*_*τ*_ = 200. For spanwise forcing to be a practical method of drag reduction, the same benefits must occur at much higher Reynolds numbers. Existing work suggests that the drag reduction decreases with increasing Reynolds number. This relationship was at first considered as a power-law [21] with the exponent a function of the forcing parameters [17], and also Reynolds number [22]. This non-physical argument has been superseded by that of Gatti and Quadrio [23] who, using analogies to wall roughness and riblets, produced an analytical model to predict drag reduction at higher Reynolds numbers. Their model provides a nondimensional relation between the vertical shift in the log-law mean velocity profile, *Re*_*τ*_ and the coefficient of friction *C*_*f*_,

2 $$ C_{f}=\frac{2\tau_{x}}{\rho U_{\infty}^{2}}=\frac{2 u_{\tau}^{2}}{U_{\infty}^{2}}.  $$

Using this relation, and existing DNS data, a potential drag reduction using travelling waves at *Re*_*τ*_ = 10^5^ of 30*%* is predicted.

Although the outlook for drag reduction with travelling waves is promising, achieving it in practise is highly challenging. It has been successfully implemented in pipe flow using water by Auteri et al. [24] who drove 60 independent azimuthally rotating sections of pipe to discretise travelling waves, with an *Re*_*τ*_ ≈ 160. The sections of pipe were connected via belts to motors which allowed 3 different wavelengths to be produced. The frequency and wavelength of the forcing were then varied, and the changes in skin friction were measured as changes in pressure loss along a length of the pipe. Auteri et al. recorded a peak drag reduction of 33*%*, with similar trends to the numerical work of Quadrio et al. [17]. They also found that upstream-travelling waves invariably produced a drag reduction, but when the velocity of a downstream-travelling wave matched the convection velocity of the near-wall structures, the skin-friction drag increased.

## Experimental Method

The majority of experimental work to date has focused on wall oscillations, with the exception being that of Auteri et al. [24] who produced travelling waves in pipe flow. The generation of streamwise travelling waves in a finite flat surface is challenging. The surface must oscillate, and do so such that for a given wall velocity, the maximum displacement is inversely proportional to the frequency of the oscillation. Consequently, generating waves of low frequency results in a low velocity of the oscillating wall. As drag reduction is known to scale monotonically with the wall velocity [17], waves with low frequency are likely to have limited impact on the flow, and even have an adverse effect [9]. Another difficulty arises in creating waves of short length in the streamwise direction, with a large displacement amplitude in the spanwise direction, as large shear strains in the surface are created. The largest streamwise wavenumbers possible in a continuum are therefore dictated by the mechanical and structural properties of the surface. To facilitate displacements of this kind (those with a short wavelength) in an elegant and efficient way, a novel surface was conceived which exploits the favourable properties of compliant structures.

### Actuator design

**Fig. 3:**
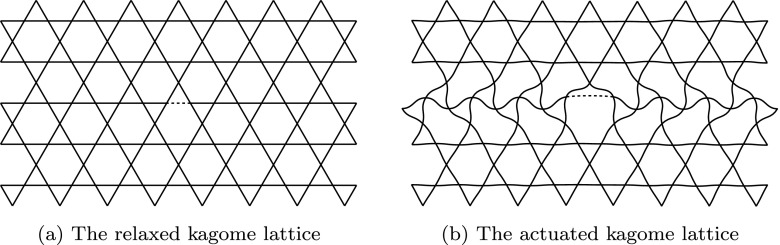
The kagome lattice shown relaxed and actuated (Wicks and Guest [25]). A single member, indicated by a dashed line, is elongated and the resulting deformation is constrained within a bounded ‘corridor’ collinear with the actuated member

**Fig. 4:**
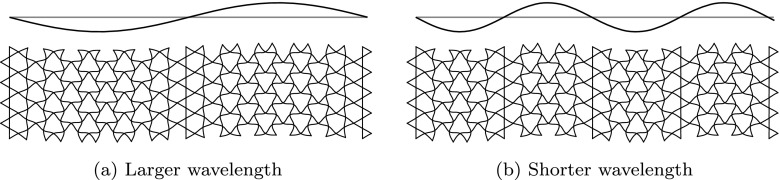
An illustration of how the planar kagome lattice can be used to discretise waveforms of varying length when the independent ‘corridors’ of deformation in Fig. 3 are driven in sequence

Mechanical engineering problems, such as the design of an active surface, are typically tackled with a large number of joints, actuators, bearings and sliders [12]. This approach is not suitable for generating travelling waves efficiently and instead the same effects were achieved in a novel way using a compliant structure based on the kagome lattice geometry. The kagome lattice has long been identified as a structure with unique structural properties which lends itself to this application – the production of in-plane travelling waves [26, 27]. When a single member of the lattice is extended, indicated as a dashed line in Fig. 3, the structural deformations are confined to a ‘corridor’ region collinear with the actuated member. The repetitive nature of the lattice means that these independent regions can be driven together, as illustrated in Fig. 4, where adjacent corridors are actuated with variable displacement to create waveforms. To produce dynamic waveforms, these corridors are excited with the same amplitude, but with a phase delay between them. By varying the phase and frequency of actuation independently, in-plane travelling waves of varying length and speed can be produced.

To provide a smooth continuous surface, the compliant kagome structure is covered with a membrane skin cast from Ecoflex 00-50 silicone rubber, 350 μm thick. The membrane is pretensioned in both directions by 30*%* before being adhered to the lattice in order to prevent it from buckling as the structure deforms. This skin spans the corridors, allowing a degree of interpolation between the actuated regions, creating a continuous velocity field at the wall. The wall forcing generated is highly dependent on the structural performance of the lattice, which necessitates its careful design to match the scaling properties of the boundary layer. The detailed design, testing and optimisation of the compliant surface is beyond the scope of this paper, but can be found, along with novel, general methods for the design of compliant structures, in other recent publications [28–30]. The structural aspects of the surface can be considered as discrete and independent areas of wall oscillation, analogous to the sections of rotating pipe in the work of Auteri et al. [24]. The membrane skin then covers these regions to create a continuous flat surface.

The design of the experiment was determined from the work of Quadrio, Ricco and Viotti [17] who produced a ‘map’ of drag reduction for varying forcing parameters: streamwise wavenumber $\kappa _{x}^{+}$ and frequency *f*^+^, a region of which is displayed in Fig. 2. A combined fluids/structures objective function was produced which adjusted the dimensions of the structure to maximise the scope of the corresponding fluids investigation at the highest *Re*_*τ*_ possible. The structure was designed to reject out-of-plane deformations, to minimise structural fatigue, and to be of a size which was physically manageable and easy to manufacture [28–30].

**Fig. 5:**
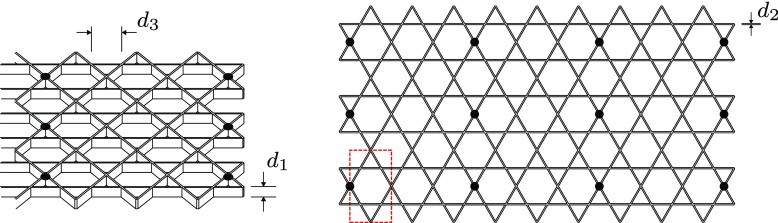
The parametrised kagome lattice structure. The location of supports is shown with black circles, and a unit cell of the geometry is indicated by the red dashed box

Large deformations are required to achieve the large wall velocities necessary to significantly influence the boundary layer [17]. This is achieved structurally with slender members which bend easily. However, this results in a sparse lattice with large distances between controlled areas of the surface, limiting the maximum streamwise wavenumber that can be produced. The minimum streamwise wavelength is dictated by the internal dimensions of the kagome structure, and can be expressed as $2\sqrt {3}d_{3}$, where *d*_3_ is the length of a single member, as illustrated in Fig. 5. Balancing these structural considerations, the maximum stresses from structural finite element simulations, and the expected boundary layer characteristics from empirical relations [31] results in a structure with the dimensions shown in Table 1. The corresponding range of wall forcing parameters consistent with the scaling of the boundary layer is displayed in Table 2. The total potential scope of the experiment is therefore illustrated in Fig. 2, as the two blue rectangles, overlaying the results reported by Quadrio and Ricco [17].

**Table 1:** Dimensions of the structure as parameterised in Fig. 5

out-of-plane height $\left (d_{1}\right )$	in-plane width $\left (d_{2}\right )$	length $\left (d_{3}\right )$
5 mm	30 μm	16 mm

**Table 2:** Boundary layer parameters. $U_{\infty min}$ and $U_{\infty max}$ give the range of freestream velocities which produce boundary layers suitable for control by active surface

$U_{\infty min}$	$U_{\infty max}$	*R* *e*_*τ*_	freq. max	$\kappa _{x}^{+}$ max	*λ* min
5 ms^− 1^	8 ms^− 1^	1000	70 Hz	0.0069	61.5 mm

With the structure designed, pneumatic air-cylinders (double acting CJ1B4-10U4 actuators manufactured by SMC) were chosen to drive the surface. They were selected for their ability to produce sufficient force and generate sizeable displacements with a high-frequency response, measured to be 8 mm peak-to-peak at 70 Hz. The actuators were controlled via VQ110 solenoid valves, powered by a custom amplifier and controlled with a National Instruments PCIe-7842R FPGA board.

### Spatial transients

Simulations show that there is a well-characterised temporal transient between the spanwise wall oscillations and their influence on the streamwise wall shear stress [7, 32, 33]. Whilst the spanwise flow adapts quickly to the oscillation, typically reaching equilibrium after one cycle, the streamwise component takes longer to reach a steady-state. This duration is independent of the forcing frequency, but scales with the square-root of the forcing velocity [33]. The temporal transient is present in developing boundary layers in the form of a spatial transient, and is a likely source of the disparity between experimental and computational drag reduction measurements in literature [14, 15, 18–20, 24, 34]. To address this, the forcing needs to take place over a significant streamwise fetch.

As a rule of thumb, the streamwise extent of an adjustment to a perturbation is ten large-eddy turnover times. For travelling surface waves, the length of this spatial transient (the response to the forcing) for a boundary layer has been measured in DNS by Skote [18, 19] to be approximately 100 times the boundary layer displacement thickness, or twice the spanwise amplitude of the forcing. This is significantly shorter than the equivalent spatial transient estimated from the transient time of *t*^+^ ≈ 1200 observed in channel flow by Quadrio and Ricco [33]. To convert the temporal transient to a spatial one, the near-wall convective velocity can be taken as approximately 10*u*_*τ*_ [35], and the resulting length is approximately 12,000 viscous units. The larger temporal transient observed by Quadrio and Ricco [33] was used to provide a conservative estimate for the required fetch,

3 $$ d_{f}=\frac{10 t^{+} \nu}{u_{\tau}} $$

where *u*_*τ*_ can be estimated from the empirical relationship for the skin-friction coefficient, [31], as

4 $$ C_{f}= 0.0576 Re_{x}^{-0.2}, $$

where $\displaystyle {Re_{x}=\frac {x U_{\infty }}{\nu }}$. A conservative estimate of *u*_*τ*_ = 0.2 ms^− 1^ yields a length of *d*_*f*_ = 1.1 m in air. This represents the required minimum streamwise fetch of actuated surface.

### Test rig

**Fig. 6:**
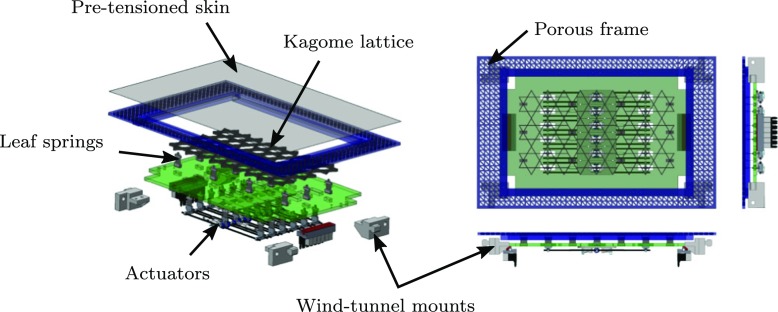
CAD model of a single wind tunnel module

**Fig. 7:**
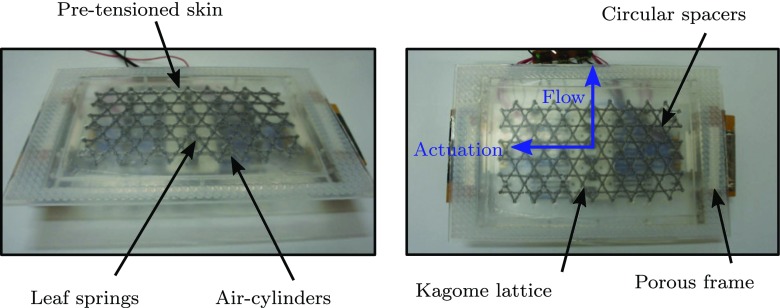
Photograph of the finished module before mounting in the wind tunnel

**Fig. 8:**
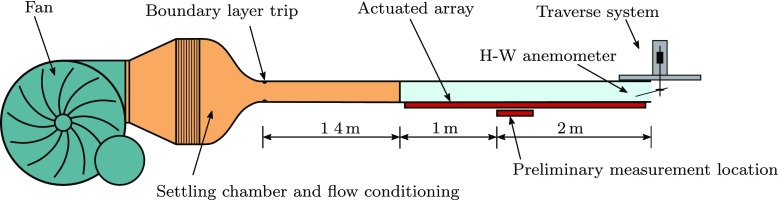
Schematic diagram of the wind tunnel, illustrating the two measurement configurations

To facilitate manufacture and testing, the structure, actuators and control electronics were assembled in modules. A CAD model illustrating the various parts of a single module is displayed in Fig. 6, and shown assembled in Fig. 7, complete with the pre-tensioned silicone surface. Two studies were conducted, the first used a single module, whilst the second used a longer array to mitigate the aforementioned spatial transients. The experimental setup is illustrated in Fig. 8. In both cases the modules were attached to base of the wind tunnel forming the floor. A centrifugal blower wind tunnel was used with a cross section of 127 × 762 mm. The boundary layer was tripped shortly after the contraction on both the upper and lower surfaces with wire 2 mm in diameter fixing transition. At the flow velocities considered, there is no clear freestream as the developing boundary layers from the ceiling and floor meet in the centre of the wind tunnel section, and therefore the terms ‘centre line’ and ‘freesteam’ are synonymous. As the channel is far from being fully developed, and the condition remains the same for the forced and unforced cases, it is unlikely that the slight interaction in the outer region of the two boundary layers will affect the overall results. Static pressure measurements were taken along the length of the section, and the side walls of the wind tunnel were flared outwards to provide a zero pressure gradient along the working section.

**Fig. 9:**
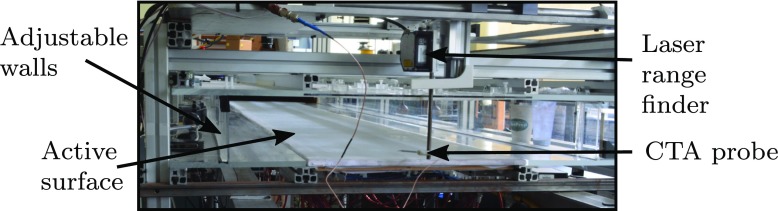
A view looking upstream from the end of the working section

**Fig. 10:**
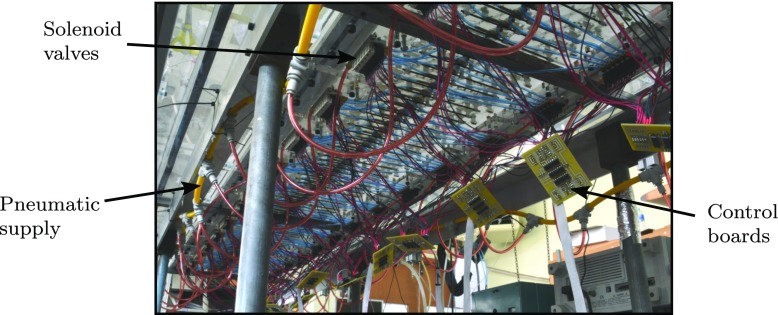
A view of the underside of the actuated surface

Figure 9 is a view looking upstream showing the deforming side walls as well as the 2.9 m long active surface formed from the 17 modules. The pre-tensioned skin, 0.35 mm thick, is also visible adhered to the structure. Also shown is a three-axis traverse for the positioning of a single hot-wire probe. The traverse was fitted with an opto NCDT 2200-2 laser range finder with a 30 nm resolution to precisely record the wall-normal displacements, mitigating any backlash in the traverse gear. The view of the underside the active surface is shown in Fig. 10, where the valves, actuators and control boards can be seen.

Single-wire constant temperature anemometry (CTA) measurements were taken with and without forcing to assess changes to the streamwise boundary layer, along with photogrammetric measurements of the surface velocity to determine the nature of the forcing and also to provide a simultaneous quantitative measurement of the wall velocity at the measurement location. Any drag reduction is only relevant if it is global, but the complexity of the experiment meant that the wall shear stress could only be assessed at a single location, and then assumed to be representative of the whole surface. This assumption is reasonable as the wall motion is uniform in the spanwise direction in all cases, and is uniform in the streamwise direction when a wall oscillation is produced. When travelling waves are produced, their wavelength is smaller than the spatial transient of the forcing, suggesting that the streamwise modulation of the wall velocity is unlikely to create significant local effects.

### The nature of the forcing

While numerical studies can prescribe the velocity of the wall as a perfect boundary condition, achieving this experimentally is unlikely to occur either in laboratory experiments, or any industrial implementation of this drag reduction technique in the far future. The discrete nature of the forcing introduced by Auteri et al. [24] and the resulting high frequency harmonics, led to variations between their measured trends and those reported in numerical studies. Despite the imperfect nature of the forcing (i.e. motion other than that defined in Eq. 1), Auteri et al. still recorded a large drag reduction.

**Fig. 11:**
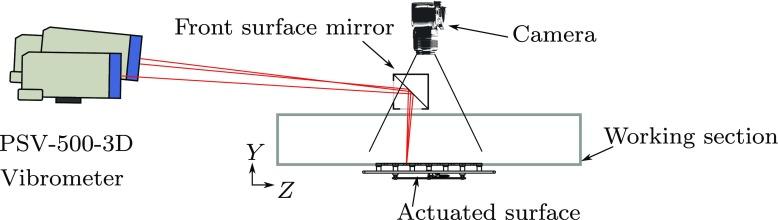
A schematic diagram showing the experimental set-up for the vibrometry and photogrammetic measurements

The velocity field generated by the kagome lattice is also not uniform, with small out-of-plane deformations. From the structural deformations shown in Fig. 3, it is clear that, although the majority of the deformation is in the direction of the actuation, the bending of the internal structure results in some out-of-plane displacement. This structural deformation naturally influences the surface motion and was characterised with digital image correlation (DIC) [30]. The surface was coated with white titanium dioxide and graphite, and filmed from above undergoing actuation at 1 kHz, using a Phantom v641 camera, as illustrated in Fig. 11. The maximum streamwise component of the deformation was found to be 20*%* of the spanwise displacement. As the structure has a membrane surface, it possesses negligible bending stiffness, and is therefore able to deform out-of-plane between the points where it is attached to the structure. The 30*%* pre-tension was selected so that the surface will not buckle as the structure deforms and also so that its resonant frequency is above the forcing frequencies required.

However, under high amplitude forcing, the surface does experience some out-of-plane displacement. This was measured with a Polytec PSV-500-3D-M laser vibrometer to be no more than 1.2 mm rms, when the surface was driven with maximum force at 40 Hz (*T*^+^ ≈ 190) with a freestream velocity of *U*_*∞*_ = 8 ms^− 1^. As a worst-case estimate, this out-of-plane displacement corresponds to 14 wall units. It was not possible to generate the unwanted streamwise and wall-normal motions independently, as they are inherent to the forcing mechanism; therefore it is not possible to quantify their effects separately. Instead, they should be seen as inevitable imperfections typical of any physical drag reduction mechanism, and a possible measure of the robustness of the present approach in generating in-plane surface waves. A full description of the design and analysis of the surface and structure is given in other recent work [28–30].

### Experimental uncertainties

The skin-friction drag coefficient was not measured directly, but instead, was inferred from the the gradient of the streamwise velocity near the wall as

5 $$ C_{f}= 2 U_{\infty}^{-2}\nu\left.\frac{\partial U}{\partial y}\right|_{y = 0},  $$

where *U* is the time-averaged streamwise velocity and *y* is the wall normal distance. Boundary layer profiles were taken without forcing to establish the baseline wall shear stress. A boundary layer profile with forcing was then recorded and further measurements were taken afterwards to ensure the baseline condition had not changed. The time between these measurements was kept to a minimum to ensure atmospheric changes were small and there was negligible change in *ν*: this was corroborated by the repeated baseline readings after the measurements with control. The wall-normal location of the hot wire was recorded with a resolution of 30 nm, and the freestream velocity *U*_*∞*_ was measured with a Pitot tube using a Furness FCO560 manometer with a resolution of 0.01 Pa, and controlled continuously using a PID control system. The main source of errors therefore comes from the hot-wire velocity measurements and the linear fitting. The fragility of the experiment meant that long sample times at each point in the boundary layer were not possible. The flow was sampled for 10 s which, at the point with largest fluctuations (*y*^+^ ≈ 20), gave an error in velocity of ± 2 mms^− 1^, based on the approach of Benedict and Gould [36], with a 95*%* confidence interval. The mean velocities are well resolved, but the curve fitting to find the gradient at the wall is a significant source of error. A t-Test of the linear fit indicates the drag-reduction *DR*(*%*) range as approximately ± 3*%* of all the values stated.

## Preliminary Results

Before a large surface was constructed, the performance of a small actuated region was assessed. A single module was placed in the wind tunnel, 2.4 m downstream from the boundary layer trip, as illustrated in Fig. 8. The wind tunnel was run with a freestream velocity of *U*_*∞*_ = 5 ms^− 1^. Boundary layer profiles were then taken on the centre line, above the end of the 152 mm-long (1900 wall units) actuated surface 2.5 m from the boundary layer trip, with a single hot-wire probe. The raw CTA measurements were band-passed filtered between 1 Hz and 15 kHz and sampled, along with the raw signal to determine the mean, at 40 kHz with a resolution of 16 bits using a National Instruments USB-6353 device.

Boundary layer measurements were taken with the surface stationary, and also with the surface producing a wall oscillation (*κ*_*x*_ = 0), at 36 Hz corresponding to a period of *T*^+^ = 110 with an *Re*_*τ*_ = 940 at the measurement location, based on the uncontrolled flow. As the actuated region is short, these values do not change appreciably over the fetch of the forcing. The motion of the wall was measured with a PSV-500-3D-M laser vibrometer, as illustrated in Fig. 11, and the pneumatic supply pressure was adjusted so that a non-dimensional spanwise wall speed of *W*^+^ = 12 was produced.

**Fig. 12:**
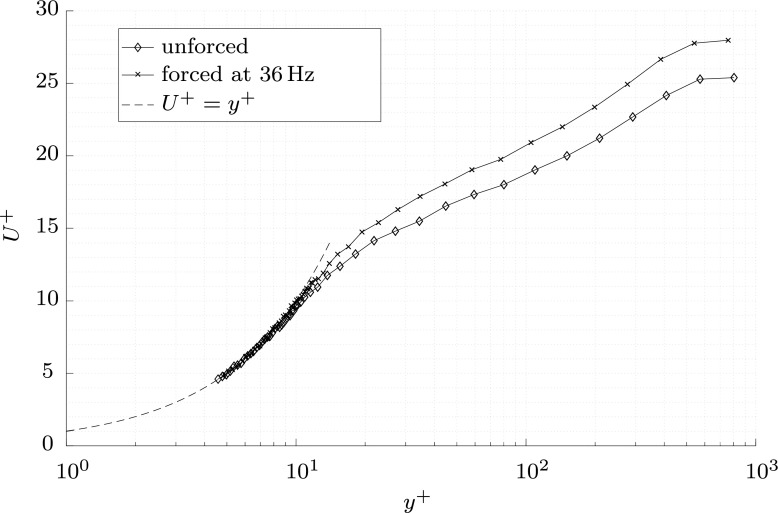
The non-dimensionalised boundary layer, with and without wall forcing, over the preliminary surface

The value of *u*_*τ*_, and an offset to the measurement height *y*, were found through an iterative linear fit of the viscous sublayer profile defined as 3.5 < *y*^+^ < 9.5. Values of *y*^+^ < 3.5 were discarded to remove any unwanted wall effects. The drag reduction, defined as

6 $$ DR(\%)=\left( 1 - \frac{C_{f,forced}}{C_{f,unforced}}\right)\times 100,  $$

was found to be 11.8*%* for this forcing. This value is lower than those recorded by others [37], but significant considering the short streamwise length of the forcing. The measured value is approximately 70*%* of the true value unaffected by the spatial transient, based on estimates from DNS at lower Reynolds numbers [37, 38]. The two boundary layer profiles, one with wall forcing and one without, are shown in Fig. 12, non-dimensionalised by their respective friction velocities and kinematic viscosity. In the linear region they collapse onto *U*^+^ = *y*^+^, but the boundary layer for the oscillating wall case is increased in the logarithmic region, confirming the observation that the viscous sublayer is thickened by the oscillations [32, 37, 38].

**Fig. 13:**
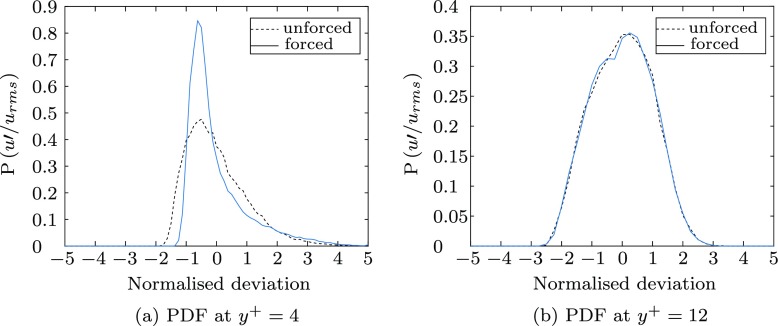
The probability density function of the fluctuating streamwise velocity, $u^{\prime }/u_{rms}$ at two heights in the boundary layer, normalised to give an area of 1

Figure 13 shows the probability density function (PDF) of the streamwise velocity fluctuations: there is a marked increase in kurtosis in the viscous sublayer (*y*^+^ = 4) and an increase in skewness, visible in Fig. 13a as a long positive tail, an indication of increased positive velocity fluctuations in this region of the boundary layer caused by the spanwise pressure gradient of the Stokes’ layer. At the edge of the viscous sublayer (*y*^+^ = 12) the distribution of fluctuations for the forced and unforced case match closely. This may be due to the combined result of the short actuation distance not allowing the Stokes’ layer to diffuse high enough into the boundary layer, and also the region being outside the influence of the Stokes’ layer penetration depth (0.9 mm). However, at the same value of *y*^+^ in other studies, a small increase in skewness and kurtosis was observed [21, 32].

The clear drag reduction displayed in Fig. 12, and the similar changes to higher moments, even after a small fetch of forcing, would justify construction of a longer dynamic surface.

## Results and Discussion

A longer surface was produced comprising 17 modules ≈ 2.9 m in length. Based on the analysis in Section 2.2, this is sufficiently long to ensure the spatial transient due to a step-change in surface condition has decayed, and so provide a large measurement region after the boundary layer has reached an equilibrium with the surface condition. The experimental configuration and measurement system was otherwise the same as that for the preliminary study. The freestream velocity was increased to *U*_*∞*_ = 6 ms^− 1^ giving *Re*_*τ*_ = 1125 at the measurement position at the end of the working section, shown in Fig. 9, 4.3 m from the boundary layer trip as illustrated in Fig. 8.

For the results presented, the nine most-downstream modules were actuated, corresponding to a length of 1.5 m (≈ 20 × 10^3^ wall units) of actuated surface before the measurement location. The pneumatic pressure supply for the actuators was adjusted to achieve a spanwise wall velocity of *W*^+^ = 12. Waves of three types were produced, indicated by the labelled black dots in Fig. 2. Point 1 corresponds to the wall oscillation case *κ*_*x*_ = 0, while point 2 corresponds to an upstream-travelling wave, $\kappa _{x}^{+}=-0.0046$, and point 3 corresponds to a downstream-travelling wave with $\kappa _{x}^{+}= 0.0046$, non-dimensioned by *u*_*τ*_ from the unforced boundary layer. The frequency of actuation was 40 Hz, corresponding to a non-dimensional time period of *T*^+^ = 100.

**Fig. 14:**
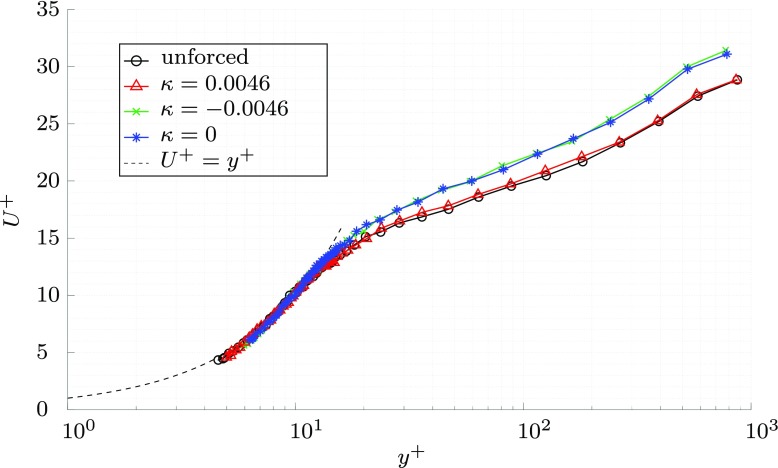
The mean velocity profile, showing the three forcing cases and the unforced case, with the 1.5 m actuated surface

The resulting boundary layer measurements are shown in Fig. 14, non-dimensionalised by their respective values of friction velocity, calculated with a linear fit of the viscous sublayer, as in the previous section. The wall oscillation and upstream-travelling wave created a drag reduction of *DR* = 19.5*%* and 21.5*%*, respectively. The downstream-travelling wave created a small drag reduction of *DR* = 2.6*%*. Like the preliminary measurements in the previous section, all the boundary layer measurements collapse in the sublayer region. The non-dimensional velocities in the logarithmic regions of the drag reducing cases are increased and the linear region is extended. The drag reduction trends match the pattern measured by Quadrio et al. [17], but the values are lower. In a DNS of channel flow, with an *Re*_*τ*_ = 1000 and the same forcing parameters, Gatti and Quadrio measured a drag reduction of *DR* ≈ 27.5*%* for the wall oscillation case and *DR* ≈ 29*%* and *DR* ≈ 7*%* for, respectively, an upstream- and downstream-travelling wave. The experimental apparatus did not allow for both accurate measurements close to the wall and hence the boundary layers shown in Fig. 14 do not quite show the full extent of the boundary layer.

The discrepancy between the two studies can be attributed to a number of factors. It is unlikely to be a result of the spatial transient as the forcing was applied over a large distance. However, it is likely to be due to the fact that the optimum non-dimensional forcing frequency *T*^+^ ≈ 100 and wavenumber are normalised by the wall shear stress, which decreases slowly in the streamwise direction in the zero-pressure-gradient boundary layer. The present surface is actuated at a single frequency, which results in values of *T*^+^,*W*^+^, and $\kappa _{x}^{+}$ which vary slowly with fetch, rather than the single value in fully developed channel flow. Using empirical relations for a turbulent boundary layer with zero pressure gradient, a conservative estimate for the range of values can be made as 100 < *T*^+^ < 150,15 < *W*^+^ < 16 and $0.0036<|\kappa _{x}^{+}|<0.0046$. Based on the DNS data of Gatti and Quadrio [23] with *Re*_*τ*_ = 1000, this is unlikely to have significant effect on the results as the corresponding changes in drag over these ranges is small. Another possible explanation is the difference in geometry between the two studies – the present work uses a developing external flow, whilst the study by Gatti et al. involves fully developed channel flow. This is supported by numerical studies by Skote [18, 19] which suggest that lower levels of drag reduction occur in boundary layers compared to internal flows. The measured drag reduction can be extrapolated to the same lower *Re*_*τ*_ = 1000 of the study by Gatti et al. [23], but this produces little change (less than 1*%*) suggesting that the difference between the studies is not caused by the small difference in *Re*_*τ*_.

The streamwise and out-of-plane motion of the wall may also affect the level of the drag reduction. Streamwise wall forcing is known to bring about a small drag reduction of 8*%* in DNS for *Re*_*τ*_ = 180 [39], whilst transverse travelling waves with an out-of-plane displacement have also produced a small drag reduction 3.4*%* [40] in a boundary layer at *Re*_*𝜃*_ = 1200. However, at higher Reynolds numbers, a drag increase was recorded. In the present experiment, there is some small out-of-plane motion (worst case, ± 1.2 mm rms) but there is no way of knowing its effect on the measured drag reduction. However, it is in-phase in the spanwise direction and so it is unlikely to produce the same changes to the flow as the transverse travelling wave produced by Roggenkamp et al. [40].

**Fig. 15:**
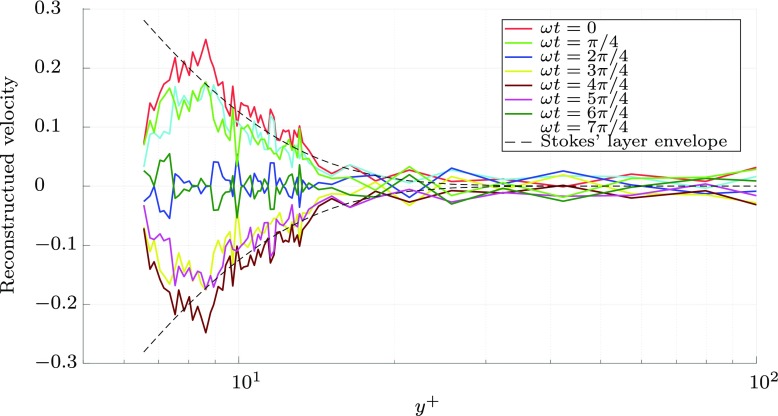
A reconstruction of the Stokes’ layer from the wall oscillation ($\kappa _{x}^{+}= 0$) forcing case, where the amplitude at 8 points in the cycle is extracted from the forcing frequency component of the velocity spectrum

The Stokes’ layer penetration depth, the distance the spanwise oscillations at the wall influence the fluid above, is a function of the frequency and velocity of the oscillating wall. The optimum wall oscillation period generates a Stokes’ layer which exclusively influences structures in the viscous sublayer. This Stokes’ layer penetration depth can be extracted from the spectrum of the streamwise velocity *u* at each measurement point, and the relative phase of the surface can be measured with the vibrometer. The amplitude and phase of measurement signal can then be used to reconstruct the influence of the wall motion, which is shown in Fig. 15 for the forcing conditions indicated by point 1 in Fig. 2. The Stokes’ layer envelope velocity, *W*_Stokes_, indicating the maximum extent of the Stokes’ layer, can be expressed as

7 $$ W_{\text{Stokes}} \propto e^{-y\sqrt{\frac{\omega}{2\nu}}}, $$

and shown as a dashed line in Fig. 15 [31]. The decay of the Stokes’ layer matches the analytical prediction closely, with the velocity decreasing significantly by *y*^+^ = 20. A single hot wire was used to measure the flow velocity. A wire of this nature is insensitive to spanwise flows and consequently the measured fluctuations are likely to be the result of the interaction of the streamwise vorticity from the wall motion, and the streamwise boundary layer, rather than the Stokes’ layer directly. As the hot wire is measuring the Stokes’ layer indirectly, a reduction in velocity is measured close to the wall, as illustrated in Fig. 15, when in practise the velocity of the Stokes’ layer tends towards the wall velocity as *y* → 0, in conformity with the no-slip condition.

**Fig. 16:**
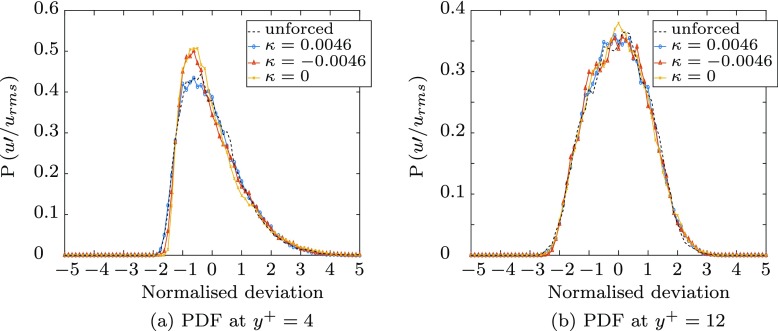
The probability density function of the fluctuating streamwise velocity, $u\prime /u_{rms}$ at two heights in the boundary layer, normalised to give an area of 1

**Fig. 17:**
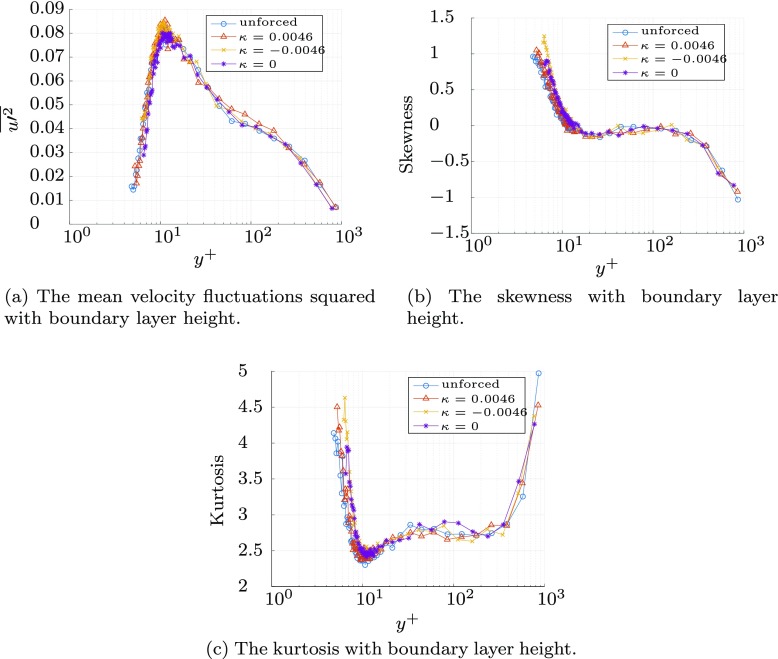
Streamwise velocity statistics for the three forcing conditions, and the unforced case, non-dimensionalised by their respective friction velocities

The change in the probability density function of the streamwise fluctuations for the longer fetch in Fig. 16 is similar but less pronounced than that for the short actuated distance, shown in Fig. 13. The upstream-travelling wave ($\kappa _{x}^{+}=-0.0046$) and wall oscillation $\kappa _{x}^{+}= 0$ cases result in the same reduction in the negative probability, and the increase in kurtosis and skewness within in viscous sublayer visible in Fig. 17. The probability at *y*^+^ = 12 exhibits similar trends, but to lesser extent, reflecting the reduced influence of the Stokes’ layer at the edge of the sublayer, as demonstrated in Fig. 15.

The wave travelling with the flow, $\kappa _{x}^{+}= 0.0046$, results in little change in the probability density function compared with the unforced case near the wall as reflected in the plots of kurtosis and skewness in Fig. 17. However, at the edge of the sublayer (*y*^+^ = 12), the streamwise travelling wave matches the other forcing cases, with a slight increase in the positive-tail and decrease in the negative-tail probability. This difference in behaviour between the near-wall and the edge of the viscous sublayer may be a result of the out-of-plane surface deformations which are dominated by the spanwise motion near the wall, but do not decay exponentially into the boundary layer at the same rate as the Stokes’ layer. The out-of-plane forcing is uncontrolled, and therefore does not exhibit the same wavespeed as the in-plane motions, and therefore influences the three forcing cases equally.

The increase in kurtosis and skewness in Figs. 17 and c in the sublayer for the largest drag reduction waves is typical of the drag reduction measured [32]. However, there is little change in the mean-square velocity measurements between the unforced case, and the different forcing conditions, as evident in Fig. 17a, where the peak fluctuations at *y*^+^ ≈ 15 stay broadly the same. This is unexpected as numerous studies have reported a decrease in turbuence intensity with forcing [14, 32]. One cause may be the out-of-plane motion driving an increase in fluctuations, despite the streamwise vorticity of the spanwise motion, and ultimate production of negative spanwise vorticity, leading to the measured reduction in velocity gradient at the wall [32]. The lack of a reduction in mean-square fluctuations in Fig. 17a suggests that the forcing is likely to be detrimental to any potential power saving, even if it results in a reduction in skin friction. The friction drag reduction typically results in a reduction in the fluctuations in the boundary layer [32] and an associated decrease in dissipation in the flow. However, as a drag reduction is observed with no reduction in the mean-square fluctuations (Fig. 17a), it is likely that the out-of-plane motion is responsible.

## Energy Budget

The actuators and the structure were not selected for efficiency of operation, but rather designed to produce a system capable of working at a range of frequencies and amplitudes. A resonant system, similar to the apparatus employed by Gouder et al. [14], would consume less power, but have to operate at a specific frequency. Based on the speed of the actuation, and the forces generated by the actuators [30], the power consumption per streamwise length of actuated surface can be calculated as 503 Wm^− 1^. Clearly, any potential power saving will be negated by the method of actuation used here.

An approach routinely employed is to assume an ideal actuator, with the only losses being the work done against the surface friction [17, 24, 38]. Achieving a meaningful measurement of the power balance was not the focus of the current work: to gain an insight of true power consumption, simultaneous measurements of the three-dimensional motion of the surface and the boundary layer need to be performed over an area, and the spanwise wall shear stress also recorded. The influence of the spatially varying wall velocity on the shear stresses at the wall needs therefore to be better characterised to calculate any net power saving. These measurements would most likely involve stereo particle image velocimetry and will form the focus of future work. These planar measurements will give the spatial distribution of the wall shear stress, ensuring any drag reduction or power saving measured is global.

## Conclusions

This paper has presented a novel way of producing in-plane travelling waves for turbulent boundary layer control in a wind tunnel experiment. Two surfaces were produced, a short preliminary surface and as well as a longer one designed to accommodate the spatial transient effects of the forcing, which is evident in the difference between the measured drag reduction for the short and long surfaces of 11.8*%* and 19.5*%* respectively, for very similar forcing parameters and flow conditions.

Changes in drag were determined by changes in the velocity gradient of the viscous sublayer using a hot wire. Data for *y*^+^ < 3.5 were not used. The larger fetch produced a maximum drag reduction of 21.5*%*, with a upstream-travelling wave ($\kappa _{x}^{+}=-0.0046$), and 19.5*%* for a wall oscillation case ($\kappa _{x}^{+}= 0$). A downstream-travelling wave ($\kappa _{x}^{+}= 0.0046$) produced a much lower reduction in drag of 2.6*%*, matching the trends reported for simulations [17, 23] and experiments [24]. The discrepancies in the absolute values of drag reduction were attributed to the imperfect nature of the forcing (the wall motion generates a small out-of-plane component equivalent to approximately 14 wall units, worst case), a slightly higher *Re*_*τ*_ = 1125, and the lower level of drag reduction observed in external flows [20]. The skewness and kurtosis of the fluctuating streamwise velocity were increased in the sublayer, typical of the drag reduction mechanism explored [32], but the mean-square velocities were unchanged: this is possibly indicative of increased turbulence production caused by the out-of-plane motion of the wall.

In spite of the imperfect nature of the forcing, a significant drag reduction was produced, with similar trends to existing work, indicating a robustness in the surface design. Future work will study the effect of forcing over an area to determine the distribution of wall shear stress and the contribution of the different components of the wall motion. In this way, an accurate assessment of the energy budget can be undertaken. A novel active surface has been shown experimentally to be capable of producing waveforms of variable length and speed which have been shown to produce a significant reduction in skin friction.
